# The effect of continuous passive motion and sling exercise training on clinical and functional outcomes following total knee arthroplasty: a randomized active-controlled clinical study

**DOI:** 10.1186/1477-7525-12-68

**Published:** 2014-05-09

**Authors:** Anett Mau-Moeller, Martin Behrens, Susanne Finze, Sven Bruhn, Rainer Bader, Wolfram Mittelmeier

**Affiliations:** 1 Department of Orthopedics, Doberaner Strasse 142, Rostock 18057, Germany; 2 Department of Exercise Science, University of Rostock, Ulmenstrasse 69, Rostock 18057, Germany

**Keywords:** Continuous passive motion (CPM), Postoperative physical therapy, Range of motion, Rehabilitation, Total knee arthroplasty (TKA)

## Abstract

**Background:**

The parallel-group randomized active-controlled clinical study was conducted to compare the effectiveness of two in-hospital range of motion (ROM) exercise programs following total knee arthroplasty (TKA). Continuous passive motion (CPM) is frequently used to increase ROM and improve postoperative recovery despite little conclusive scientific evidence. In contrast, a new active sling-based ROM therapy requires the activation of the knee joint muscles and dynamic joint stabilization. It was hypothesized that higher demands on muscle strength and muscle coordination during sling exercise training (ST) might be advantageous for early recovery following TKA.

**Methods:**

A total of 125 patients undergoing primary TKA were assessed for eligibility. Thirty-eight patients were randomly assigned to receive ST or CPM (control intervention) during hospital stay. Patients were assessed before TKA for baseline measurement (pretest), 1 day before discharge (posttest) and 3 months after TKA (follow-up). The passive knee flexion range of motion (pFL) was the primary outcome measure. Secondary outcome measures included active knee flexion range of motion, active and passive knee extension ROM, static postural control, physical activity, pain, length of hospital stay as well as clinical, functional and quality-of-life outcomes (SF-36, HSS and WOMAC scores). Data were analyzed according to the intention-to-treat principle. Differences between the groups were tested for significance by the unpaired Student’s *t* test or an analysis of covariance (ANCOVA) adjusted for baseline, weight, sex, age, pain and physical activity.

**Results:**

A between-group difference could be determined at posttest. The pFL was significantly higher by 6.0° (95% CI 0.9 to 11.2°; *P* = 0.022) in the ST group. No difference between groups in pFL was documented at follow-up. Furthermore, no significant differences could be observed for any secondary outcome measure at posttest and follow-up.

**Conclusions:**

ST seems to have a clinically relevant beneficial short-term effect on pFL compared to CPM. The results support the implementation of ST in rehabilitation programs following TKA.

**Level of evidence:**

Therapy, level 2b

## Background

The major objectives of rehabilitation after total knee arthroplasty (TKA) are the early regain of range of motion (ROM) and mobilization of the patient. Continuous passive motion (CPM) has been frequently used as part of the postoperative care regime following TKA with the aim to increase knee joint mobility and improve postoperative recovery despite little conclusive scientific evidence [1-4]. Conflicting research findings have generated an ongoing debate on its usage. The evidence for the effects on pain [5,6], function [5,7], length of hospital stay [8,9], swelling [6,10] and quadriceps strength [5,11] is inconclusive. The Cochrane Review of Harvey et al. reported evidence for small short-term effects of CPM on active and passive knee flexion ROM (aFL, pFL) of 2° and 3° [1]. The ROM is a primary indicator of a successful TKA [12] and is directly related to function [13]. Adequate knee flexion up to 90-120° is required for activities of daily living such as sit to stand transfers and climbing stairs [13]. Consequently, most research on the effectiveness of CPM focuses on ROM as the primary outcome variable [5,7,14-18]. The attainment of at least 0-90° ROM is the goal upon hospital discharge and a more functional range of 0-120° should be attained upon completion of postoperative physiotherapy. Nevertheless, Harvey et al. suggested that the beneficial effects of CPM on ROM are too small to be practically relevant. Clinically meaningful differences between standard physiotherapy and standard physiotherapy combined with CPM are reported to be at least 5° [1].

As the greatest loss of function occurs in the first month following TKA [19], it is surprising that the ROM therapy during hospital stay is still carried out passively. A passive mobilization of the knee joint with CPM does not encourage the patients to actively participate in their rehabilitation. Research on the effectiveness of active ROM exercises added to standard physiotherapy during the short in-hospital period is lacking so far. Only two studies have investigated an adjunctive active motion therapy by comparing it to patients treated with physiotherapy plus CPM and to patients treated only with physiotherapy [17,20]. Group differences were not reported, indicating that an adjunctive active ROM therapy has no benefit for patients’ recovery. However, it should be taken into account that knee joint mobilization exercises using passive or active motion machines are guided movements and are therefore less functional.

The present randomized clinical study was conducted to compare a new active sling-based in-hospital ROM exercise program with the standard-of-care therapy (CPM) following TKA. Sling exercises are self-induced and non-guided movements with unstable support which require the activation of muscles and dynamic knee joint stabilization. It was assumed that higher demands on muscle strength and muscle coordination during ST might be advantageous for early recovery following TKA

Whether an early postoperative application of ST could be beneficial for postoperative ROM, pain, physical activity, static postural control, length of hospital stay as well as clinical, functional and quality-of-life outcomes compared to CPM therapy was analyzed.

## Methods

The two arm parallel-group superiority randomized active-controlled clinical study was conducted from January 2011 to April 2012 and approved by the Ethical Review Commitee of the University Medicine Rostock (A 2009 25).

Eligible participants were patients undergoing primary TKA for osteoarthritis aged 50 to 80 years with a body mass index (BMI) less than 40. Patients with contralateral TKA or total hip arthroplasty were included when the surgery was performed more than one year before the current TKA. Exclusion criteria were as follows: musculoskeletal and neurological disorders that limit physical function, any planned further joint surgery within 12 months and substantial pain or functional limitation which made the patients unable to perform the study procedures. Prior to participation, written informed consent was obtained from all participants. Afterwards, eligible patients were randomly assigned to one of two treatment groups using blocked randomization by a computer-generated table of random numbers, a block size of ten and an allocation ratio of 1:1. Participants were sequentially allocated to the treatments in the order in which they were recruited. Intervention assignment was ascertained using sealed, opaque envelopes with consecutive numbering after the enrolled patients completed all baseline measurements. The investigator who opened the envelopes and carried out the implementation of assignments was not involved in the generation and allocation concealment. Outcome investigators and participants were blinded to the treatment at baseline measurements. Afterwards, participants and physiotherapists were aware of the group allocation due to the nature of the intervention.

All patients underwent a standard surgical procedure by inserting the same implant (Multigen Plus, Lima-Lto, San Daniele, Italy) with an identical surgical approach. Postoperatively they received continuous peridural analgesia or femoral nerve block. Additionally, a 3-step analgesia was performed to provide optimal pain relief with (1) indomethacin (25 mg), (2) metamizol and (3) paracetamol. The Multigen Plus implant is a non-constrained surface replacement consisting of symmetrical, cruciate-retaining, cemented metallic femoral and tibial components and fixed-bearing ultra-high molecular weight polyethylene liners. All patients underwent full-weight-bearing with two crutches beginning on the second postoperative day.

### Interventions

Eligible patients were either allocated to (a) the CPM group, which received physiotherapy and CPM application (control intervention; standard-of-care therapy) or (b) the ST group, which received physiotherapy and performed sling exercises. All patients participated in a standardized in-hospital physiotherapy which was carried out by physical therapists twice a day for 30 minutes each, starting on the first postoperative day. Physiotherapy consisted of active and passive ROM exercises, active isometric contractions of the quadriceps and exercises to improve activities of daily living like transfer from bed to chair, transition from sitting to standing, walking and climbing stairs. Exercise intensity was gradually increased according to pain and tolerance. Furthermore, patients received two 30 minutes CPM or ST applications each day from the second postoperative day until 1 day prior to discharge. The patients were shown the CPM or the ST exercises by a physiotherapist.

The CPM protocol was started with 0° to the maximum tolerated flexion at the highest, adjustable speed. ROM was increased daily depending on tolerance. The CPM machines used were Kinetec® Optima^TM^ S3 and S4 (AbilityOne Kinetec S.A., Tournes, France) with maximal possible flexion angles of 115° and 120°. Participants were instructed not to resist or actively support the motion of the device.

**Figure 1:**
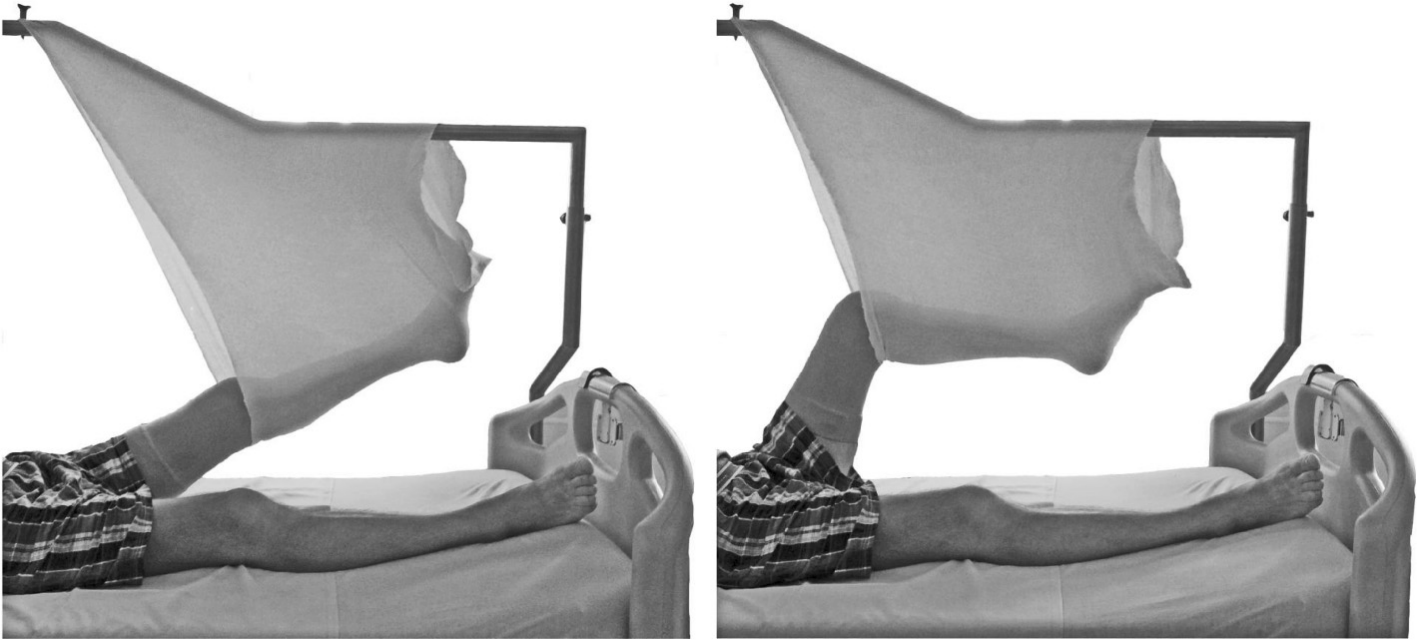
Sling exercise training.

The participants in the ST group performed active knee flexions and extensions in a sling while lying in a supine position. The sling exercise intervention is shown in Figure 1. The patient’s leg was placed in a standard tubular bandage that was suspended from a cross brace fixed to the bed. The ST protocol was started with 0° to maximum tolerated flexion at a movement speed comparable to those used in the CPM protocol. Exercise progression was achieved by asking the patients to gradually increase the range of motion as tolerated.

Patients were discharged when sufficiently mobile (i.e., at least 90° knee flexion and no need of personal care) and medically stable. After discharge, all patients participated in daily physical therapy for 3 weeks in a rehabilitation hospital.

### Outcomes

Participants were assessed before TKA for baseline measurement (pretest), 1 day before discharge (posttest) and 3 months after TKA (follow-up). The pFL was the primary outcome measure. Secondary outcome measures included aFL as well as active and passive knee extension ROM (aEX, pEX), static postural control, physical activity, pain and length of hospital stay. Furthermore, clinical, functional and quality-of-life outcomes were evaluated (i.e., SF-36, HSS and WOMAC scores). Any outcomes were determined by the same investigator. Table 1 shows the methods and parameters.

**Table 1:** Primary and secondary outcomes

**Variable**	**Method**
***Primary outcome***	
pFL	Goniometry
***Secondary outcomes***	
aFL	Goniometry
aEX	Goniometry
pEX	Goniometry
Physical activity	Activity monitor
Static postural control	Force plate
Length of hospital stay	
Pain	Visual analogue scale
Clinical, functional and quality-of-life outcomes	HSS, SF-36 and WOMAC scores

*Abbreviations*: pFL, maximal passive knee flexion; aFL, maximal active knee flexion; aEx, maximal active knee extension; pEx, maximal passive knee extension; HSS score, Hospital for Special Surgery Score; SF-36, Short Form Health Survey; WOMAC score, Western Ontario and McMaster Osteoarthritis Score.

#### Range of motion (ROM)

Active and passive ROM (aFL, pFL, aEX and pEX) were measured using a standard handheld goniometer with the patient in a supine position [21]. The goniometer was placed over the joint space with one arm in line with the fibular head and lateral malleolus and the other aligned with the greater trochanter. The knee was flexed maximally and the angle was measured in degrees. Knee extension was measured in the same position as for flexion. The knee was moved to maximal extension and the angle was measured. Jakobsen et al. (2010) showed substantial inter-tester and intra-tester reliability of the knee joint ROM measurement in patients with TKA [22].

#### Pain

Pain was evaluated using a 10 cm visual analog scale (VAS). Patients were asked to mark their degree of knee pain on a continuous horizontal line whereby the very left end indicated no pain (score 0) and the very right end (score 10) indicated unbearable pain. Studies have shown that the VAS is more reliable than the questionnaires in patients with TKA [23].

#### Physical activity

Physical activity was determined with an accelerometer-based mobility monitoring device (activPAL^TM^, PAL Technologies Ltd., Glasgow, UK) [24]. The activity monitor was attached to the thigh anteriorly in the middle between the knee and the hip. Physical activity was monitored during hospital stay over a period of 5 days (fourth to eighth day) and 3 months postoperatively for 7 days. During hospital stay the sensor was applied to the thigh of the un-operated leg to preclude measurement errors due to intervention-related joint mobilization. At the follow-up the sensor was fixed to the right thigh. Participants were instructed to wear the device permanently except when performing water activities (e. g., taking a shower, swimming). The absolute time spent lying/sitting, standing and stepping as well as the number of steps and sit to stand transitions were measured. Data were obtained with a sampling frequency of 10 Hz and analyzed with the activPAL^TM^ interface program.

#### Static postural control

The participants executed postural tasks in upright bipedal stance on a force plate (GKS 1000®, IMM Holding GmbH, Mittweida, Germany) in static condition. The force plate measured the trajectory of the center of pressure (CoP) in medio-lateral (m-l) and anterior-posterior (a-p) direction. Participants were asked to stand as stable as possible, in slight knee flexion and with hands akimbo. The position of feet was marked for repeated testing. Two conditions of vision were tested: (1) eyes open, (2) eyes closed. In the eyes-open condition, the participants were instructed to fixate a black point (Ø 10 cm) located 1.20 m away from the platform at eye level. In the eyes-closed condition, they were instructed to close their eyes and maintain the gaze straight ahead. In order to become familiar with the procedure the participants completed three pre-trials. Thereafter, three test trials each of 15 seconds duration were performed. Before the recording of measurement was started, patients had to stabilize stance for 10 seconds. A rest period of 1 minute was allowed between the trials. Data were obtained with a sampling frequency of 40 Hz and analyzed with the GKS-MED Software (IMM Holding GmbH, Mittweida, Germany) und Excel 2007 (Microsoft Inc., Seattle, USA). The velocity and sway of the CoP displacement in m-l and a-p direction (velocity_m-l_, velocity_a-p_, sway_m-l_, sway_a-p_) and the area of the CoP displacement were evaluated. The mean value of the three test trials was used for further analyses.

#### Clinical, functional and quality-of-life outcomes

Health-related status, function and quality of life were evaluated using the 36-item Short Form Health Survey (SF-36) [25], Hospital for Special Surgery Knee Score (HSS) [26], and Western Ontario and McMaster University Osteoarthritis Index (WOMAC) [27]. The scores are generally acknowledged to have good reliability and validity [25,27,28]. Each scale ranges from 0 (poorest status) to 100 points (best status). The SF-36 consists of 36 items assigned to 8 subscales: physical functioning, physical role, bodily pain, general health, vitality, social functioning, emotional role and mental health. Two summary scores (mental and physical health) and one total score were calculated as the mean of the 8 subscales. The WOMAC score is a disease-specific, self- administered, health status measure using 3 subscales with a total of 24 items: pain (5 items), function (17 items) and stiffness (2 items). Each question was answered using a 5-point Likert scale. The HSS score is subdivided into six categories: pain, function, range of motion, muscle strength, flexion deformity and instability.

### Statistical analysis

There are no preliminary studies on the comparison of both interventions. Based on the review of Harvey et al. (2010) and with the aim of showing clinically relevant differences, we hypothesized a difference in ROM between ST and CPM therapy of 5° [1]. Sample size calculation indicated that a total of 52 patients would be required to detect a large effect (Cohen’s *f* = 0.40) with a two-sided significance of 0.05 and a power of 0.80. In considering an anticipated dropout rate of 10%, a total of 58 patients were needed for the trial. A 14-month recruitment period was assumed to enroll this number of participants.

Data analysis included all randomized patients according to their original treatment allocation (intention-to-treat analysis) [29]. Data were checked for normal distribution using the Shapiro-Wilk *W* Test. Non-normally distributed data were log-transformed before analysis. Multiple imputation (5 imputed data sets) was used to account for missing data using the Markov Chain Monte Carlo (MCMC) method [30]. Differences between the groups were tested for significance by the unpaired Student’s *t* test (*P* ≤ 0.050) or an analysis of covariance (ANCOVA) adjusted for baseline [31], weight, sex, age, pain and physical activity (alpha-adjustment for conducting two ANCOVA’s *P* ≤ 0.050/2 = 0.025). All data were analyzed using the SPSS statistical package (version 20.0, SPSS Inc., Chicago, IL, USA). Sample size, power and Cohen’s effect size were calculated with the statistical software package G*Power (version 3.1.4.) [32]. The effect size *f* was interpreted using the classification of Cohen (1988): *f* = 0.10 small effect, *f* = 0.25 medium effect, *f* = 0.40 large effect [33].

Data of the pretest are presented as mean and standard deviation in the tables. Pooled multiple imputation data of each primary and secondary outcome are reported as covariate-adjusted means and standard deviation together with the effect size (mean difference) and its precision (95% confidence intervals, 95% CI) in the figures and tables.

## Results

**Figure 2:**
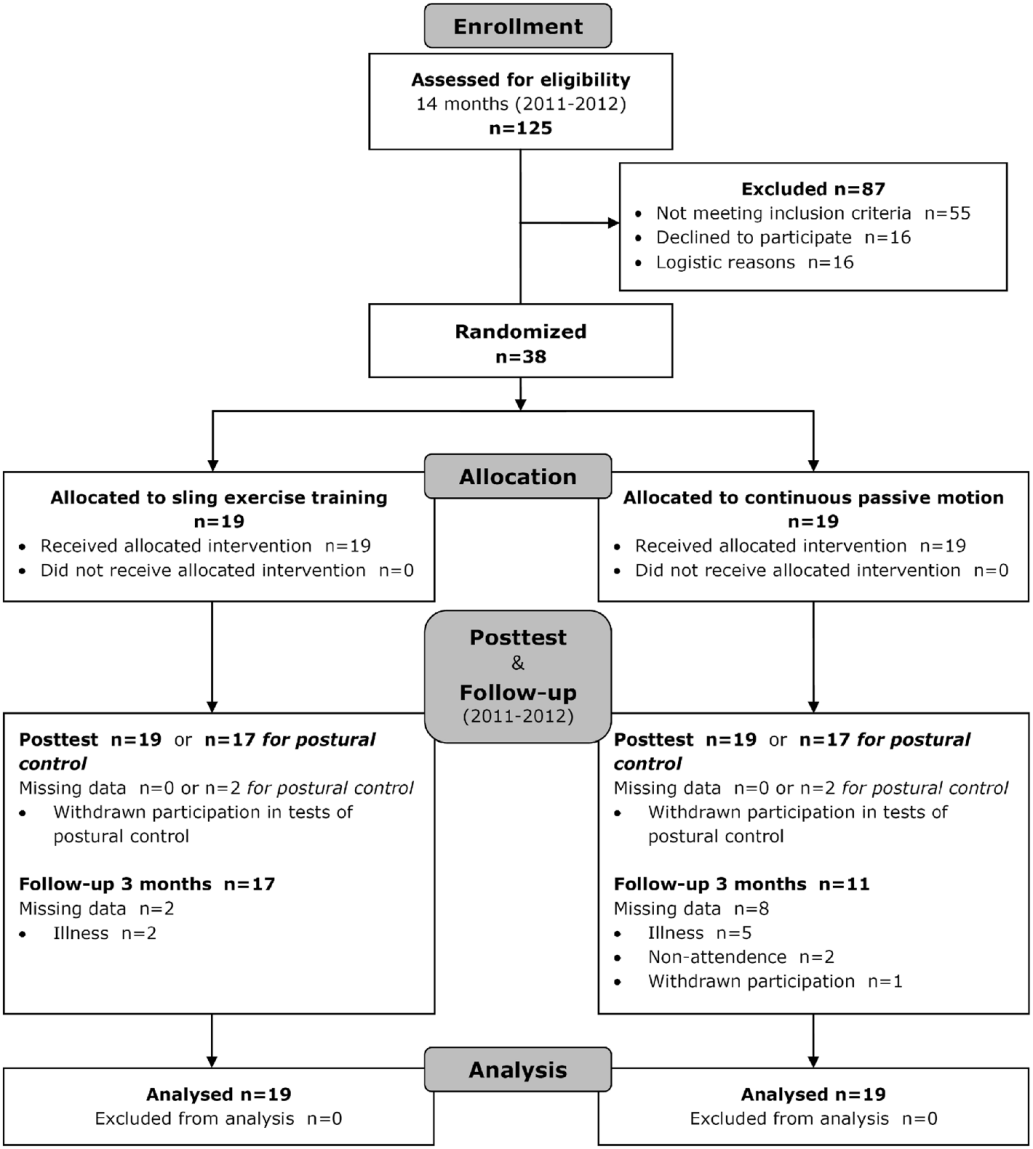
**CONSORT flow diagram to show the flow of participants through the trial **[34]**.**

Thirty-eight participants were recruited from 125 available patients within the 14 month recruitment period. The recruitment of patients was stopped when the scheduled date of closure was reached. The minimum sample size (N = 58) required was not achieved. Recruitment numbers, reasons for not being eligible or enrolled as well as allocation, missing data and reasons for missing data are reported in the CONSORT flow diagram (Figure 2). The patients’ demographic and clinical characteristics are displayed in Table 2. All patients received an allocated intervention and were analyzed for outcome measures. Both groups did not differ significantly in the number of physiotherapy, CPM and ST interventions and in the time to posttest and follow-up (Table 3). No incidents of adverse effects or harm during the study could be observed.

**Table 2:** Baseline demographic and clinical subject characteristics

**Variable**	**ST**	**CPM**
	**(n = 19)**	**(n = 19)**
Age, yrs	68.8 (8.0)	67.1 (8.8)
Sex, men	12.0 (63.2%)	10.0 (52.6%)
Weight, kg	88.9 (13.3)	93.6 (15.9)
Height, m	1.69 (0.1)	1.68 (0.1)
BMI	31.1 (4.2)	33.3 (5.1)
Affected side, right	8.0 (42.1%)	7.0 (36.8%)
Hypertension	13.0 (68.4%)	14.0 (73.7%)
Cardiac problems	6.0 (31.6%)	5.0 (26.3%)
Pulmonary problems	1.0 (5.3%)	0.0 (0%)
Diabetes	2.0 (10.5%)	5.0 (26.3%)
Cancer	1.0 (5.3%)	0.0 (0%)
TKA contralateral side	5.0 (26.3%)	6.0 (31.6%)
THA contralateral side	2.0 (10.5%)	0.0 (0%)
THA ispilateral side	3.0 (15.8%)	1.0 (5.3%)

*Abbreviations:* ST, sling exercise training group; CPM, continuous passive motion group; BMI, body mass index; TKA, total knee arthroplasty; THA, total hip arthroplasty.

Values are presented as mean (standard deviation) or numbers (%).

**Table 3:** Number of interventions during hospital stay and time to posttest and follow-up

**Variable**	**ST**	**CPM**	**Mean difference**	***P***
	**(n = 19)**	**(n = 19)**	**(95% CI)**	
CPM or ST, n	14.2 (2.2)	14.6 (1.8)	−0.46 (−1.8, 0.89)	0.597
Standard physiotherapy, n	8.1 (1.0)	8.4 (0.7)	−0.32 (0.29, −0.90)	0.281
Gait training, n	6.4 (1.3)	6.6 (1.0)	−0.26 (−1.00, 0.47)	0.472
Start day of walking corridor^‡^	4.0 (1.2)	4.3 (1.2)	−0.31 (−1.10, 0.49)	0.437
Start day of climbing stairs^‡^	7.5 (1.6)	7.5 (1.6)	<0.01 (−0.99, 0.99)	0.997
Postest, d	9.6 (1.3)	9.5 (0.6)	0.06 (−0.67, 0.78)	0.870
Follow-up, d	95.5 (9.7)	90.7 (8.7)	4.77 (−2.89, 12.44)	0.212

*Abbreviations:* ST, sling exercise training group; CPM, continuous passive motion group.

^‡^ANCOVA: adjusted for sex, weight, age, pain and physical activity.

Values are presented as means (standard deviation).

### Primary outcome

**Figure 3:**
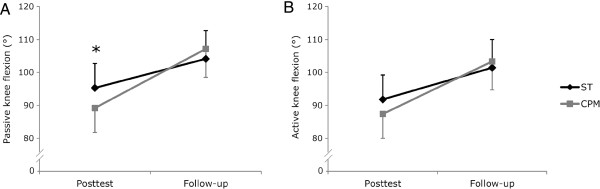
**The Graphs show the comparisons between the groups. A**. Passive knee flexion range of motion (ROM). **B**. Active knee flexion ROM. The dark grey line represents the sling exercise training group (ST) and the light grey line the continuous passive motion group (CPM). Data are presented as estimated marginal means and standard deviation (ANCOVA: posttest adjusted for baseline, sex, weight, age, pain and physical activity; follow-up adjusted for baseline, sex, weight, age and physical activity. * denotes a significant difference (*P* ≤ 0.025).

A between-group difference could be determined at posttest. The pFL was significantly higher by 6.0° (*F* = 5,80; *P* = 0.022; *η*_*p*_^*2*^ *= 0.162; f* = 0.440) in the ST group. No difference in pFL was documented at follow-up (Table 4; Figure 3).

**Table 4:** Outcome measures of knee joint range of motion and static postural control with open and closed eyes

	**Pretest**		**Posttest**^**‡**^			**Follow-up**^**‡**^		
**Variable**	**ST**	**CPM**	**ST**	**CPM**	**Mean difference**	**ST**	**CPM**	**Mean difference**
	**(n = 19)**	**(n = 19)**	**(n = 19)**	**(n = 19)**	**(95% CI)**	**(n = 19)**	**(n = 19)**	**(95% CI)**
**Range of motion**								
Active flexion, °	108.4 (15.1)	103.0 (21.7)	91.8 (6.9)	87.4 (6.9)	4.4 (−0.4, 9.1)^†^	101.4 (9.6)	103.3 (9.6)	−1.9 (−8.5, 4.7)
Passive flexion, °	111.6 (13.7)	106.1 (20.2)	95.3 (7.4)	89.2 (7.4)	6.0 (0.9, 11.2)*	104.1 (8.6)	107.1 (8.6)	−3.0 (−8.9, 2.9)
Active extension, °	4.5 (5.2)	3.4 (6.7)	1.6 (3.2)	1.7 (3.2)	−0.1 (−2.3, 2.1)	3.8 (5.4)	2.0 (5.4)	1.8 (−1.9, 5.4)
Passive extension, °	3.6 (4.0)	3.4 (5.5)	0.7 (1.6)	<0.1 (1.6)	0.7 (−0.4, 1.8)	2.8 (4.1)	0.8 (4.1)	2.0 (−0.8, 3.8)
**Postural control – eyes open**								
Area, cm^2^	1.50 (0.52)	1.47 (0.92)	1.63 (0.83)	1.62 (0.83)	0.01 (−0.54, 0.56)	1.42 (0.62)	1.44 (0.62)	−0.03 (−0.43, 0.38)
Sway_m-l_, mm	5.33 (2.46)	4.84 (1.77)	6.61 (2.73)	7.29 (2.73)	−0.68 (−2.50, 1.14)	6.25 (2.18)	6.12 (2.18)	0.13 (−1.30, 1.56)
Sway_a-p_, mm	5.66 (2.22)	4.99 (1.64)	8.77 (4.11)	7.86 (4.11)	0.92 (−1.82, 3.66)	7.29 (2.95)	6.17 (2.95)	1.12 (−0.83, 3.07)
Velocity_m-l_, mm∙s^−1^	11.32 (2.66)	11.55 (4.78)	17.87 (7.59)	18.03 (7.59)	−0.17 (−5.19, 4.86)	15.70 (8.59)	18.32 (8.59)	−2.62 (−8.26, 3.02)
Velocity_a-p_, mm∙s^−1^	12.53 (3.09)	11.89 (3.40)	24.39 (8.72)	22.39 (8.72)	2.00 (−3.80, 7.79)	19.64 (8.00)	20.13 (8.00)	−0.48 (−5.74, 4.77)
**Postural control – eyes closed**								
Area. cm^2^	3.24 (2.11)	4.16 (3.39)	3.83 (1.41)	3.23 (1.41)	0.59 (−0.35, 1.53)	3.19 (2.09)	3.68 (2.09)	−0.49 (−1.87, 0.89)
Sway_m-l_, mm	6.30 (2.08)	6.90 (2.28)	6.84 (2.55)	7.13 (2.55)	−0.29 (−1.98, 1.40)	6.36 (2.01)	6.03 (2.01)	0.32 (−1.00, 1.64)
Sway_a-p_, mm	7.33 (2.65)	8.29 (4.76)	9.12 (3.98)	7.54 (3.98)	1.58 (−1.05, 4.22)	7.99 (2.31)	7.33 (2.31)	0.66 (−0.86, 2.18)
Velocity_m-l_, mm∙s^−1^	16.54 (5.48)	19.10 (11.70)	18.61 (5.59)	17.37 (5.59)	1.23 (−2.47, 4.94)	16.95 (7.25)	17.20 (7.25)	−0.25 (−5.04, 4.54)
Velocity_a-p_, mm∙s^−1^	19.34 (5.58)	21.63 (14.36)	25.71 (6.44)	21.21 (6.44)	4.50 (0.23, 8.77)^†^	20.44 (6.66)	19.41 (6.66)	1.03(−3.36, 5.42)

*Abbreviations:* ST, sling exercise training group; CPM, continuous passive motion group; m-l, medio-lateral; a-p, anterior-posterior.

*denotes a significant difference (*P* ≤ 0.025); ^**†**^denotes a statistical tendency (*P* ≤ 0.100). ^‡^ANCOVA: values of the posttest were adjusted for baseline, sex, weight, age, pain and physical activity; values of the follow-up-measurement were adjusted for baseline, sex, weight, age and physical activity.

Values of the pretest are presented as mean (standard deviation). Values of the posttest and follow-up-measurement are presented as estimated marginal means (standard deviation).

### Secondary outcomes

No significant differences between the groups could be observed for any secondary outcome measure at posttest and follow-up (Tables 4, 5, 6). However, a statistical tendency toward an increase in aFL by 4.4° (*F* = 3.53; *P* = 0.070; *η*_*p*_^*2*^ *= 0.105; f* = 0.343; Power = 0.533) could be documented for the ST group (Table 4; Figure 3). Furthermore, the velocity_a-p_ for the eyes closed condition tended to be higher by 4.5 mm∙s-1 (*F* = 4.66; *P* = 0.040; *η*_*p*_^*2*^ *= 0.143; f* = 0.480; Power = 0.683) and the HSS score demonstrated a trend toward lower muscle strength by −0.9 (*F* = 4.20; *P* = 0.049; *η*_*p*_^*2*^ *= 0.116; f* = 0.362; Power = 0.582) in the ST group at posttest (Tables 4, 6).

**Table 5:** Outcome measures for pain and physical activity

	**Pretest**		**Posttest**^‡^			**Follow-up**^**‡**^		
**Parameter**	**ST**	**CPM**	**ST**	**CPM**	**Mean difference**	**ST**	**CPM**	**Mean difference**
	**(n = 19)**	**(n = 19)**	**(n = 19)**	**(n = 19)**	**(95% CI)**	**(n = 19)**	**(n = 19)**	**(95% CI)**
Pain	n/a	n/a	1.22 (1.73)	1.78 (1.73)	−0.56 (−1.73, 0.60)	n/a	n/a	n/a
Length of hospital stay, d			9.99 (0.31)	10.52 (0.32)	−0.53 (−1.47, 0.41)			
**Physical activity**								
Lying/sitting, h	n/a	n/a	112.0 (3.4)	111.5 (3.4)	0.5 (−1.8, 2.8)	134.4 (9.1)	132.3 (9.1)	2.2 (−4.0, 8.2)
Standing, h	n/a	n/a	7.1 (2.7)	6.1 (2.7)	1.0 (−0.8, 2.8)	25.3 (6.0)	25.5 (6.0)	−0.2 (−4.2, 3.8)
Stepping, h	n/a	n/a	1.4 (0.80)	1.3 (0.80)	0.1 (−0.5, 0.6)	9.5 (3.6)	9.4 (3.6)	0.1 (−2.3, 2.6)
Sit to stand transition, n	n/a	n/a	156.4 (56.3)	129.2 (56.3)	27.2 (−10.9, 65.2)	330.5 (102.4)	320.8 (102.4)	9.7 (−59.0, 78.4)
Step count, n	n/a	n/a	4685 (3087)	4151 (3087)	534 (−1555, 2624)	42037 (14412)	36203 (14412)	5834 (−3836, 15504)

*Abbreviations:* ST, sling exercise training group; CPM, continuous passive motion group; n/a, not available.

^‡^ANCOVA: pain was adjusted for sex, weight, age and physical activity; length of hospital stay was adjusted for sex, weight, age, pain and physical activity; the posttest-values of physical activity were adjusted for sex, weight, age and the follow-up-values were adjusted for sex, weight and age.

Values are presented as estimated marginal means (standard deviation).

**Table 6:** Outcome measures of clinical, functional and quality-of-life outcomes

	**Pretest**		**Posttest**^**‡**^			**Follow-up**^**‡**^		
**Variable**	**ST**	**CPM**	**ST**	**CPM**	**Mean difference**	**ST**	**CPM**	**Mean difference**
	**(n = 19)**	**(n = 19)**	**(n = 19)**	**(n = 19)**	**(95% CI)**	**(n = 19)**	**(n = 19)**	**(95% CI)**
**HSS score, %**								
Pain	10.8 (6.9)	14.5 (7.4)	24.3 (6.4)	22.3 (6.4)	1.9 (−2.5, 6.4)	23.1 (6.1)	20.9 (6.1)	2.2 (−2.0, 6.4)
Function	13.7 (4.1)	15.1 (4.2)	9.7 (3.4)	10.0 (3.4)	−0.3 (−2.6, 2.0)	16.7 (3.1)	17.7 (3.1)	−1.1 (−3.2, 1.1)
Strength	10.0 (0.0)	9.9 (0.5)	8.9 (1.3)	9.7 (1.3)	−0.9 (−1.7, 0.0)^†^	10.0 (0.0)	10.0 (0.0)	-
Instability	9.6 (1.2)	9.6 (1.2)	10.0 (0.0)	10.0 (0.0)	-	10.0 (0.0)	10.0 (0.0)	-
FlexDeform	9.1 (1.9)	8.7 (2.3)	10.0 (0.0)	10.0 (0.0)	-	10.0 (0.0)	10.0 (0.0)	-
ROM	13.9 (1.6)	13.1 (2.4)	12.3 (1.0)	11.5 (1.0)	0.8 (0.2, 1.5)*	12.7 (1.8)	12.9 (1.8)	−0.2 (−1.4, 1.1)
Total	66.3 (11.8)	69.9 (11.8)	71.6 (9.7)	70.8 (9.7)	0.8 (−5.8, 7.4)	81.6 (10.2)	79.2 (10.2)	2.4 (−4.5, 9.4)
**SF-36 score, %**								
Physical health	32.9 (16.2)	30.3 (13.5)	40.0 (14.0)	37.6 (14.0)	2.5 (−6.9, 11.9)	52.5 (19.7)	52.6 (19.7)	0.1 (−13.3, 13.2)
Mental health	66.3 (19.8)	57.8 (20.5)	68.1 (18.1)	66.2 (18.1)	1.6 (−10.6, 13.9)	72.7 (18.2)	69.4 (18.2)	3.2 (−9.0, 15.6)
Total	49.9 (15.1)	44.0 (15.4)	50.9 (14.3)	52.3 (14.3)	−1.4 (−11.1, 8.3)	63.5 (16.3)	61.3 (16.3)	2.2 (−8.8, 13.2)
**WOMAC score, %**								
Pain	8.9 (3.3)	9.5 (3.9)	15.2 (3.7)	14.9 (3.7)	0.3 (−2.2, 2.8)	15.2 (3.6)	14.7 (3.6)	0.42 (−2.03, 2.87)
Stiffness	4.6 (2.0)	4.0 (1.9)	6.0 (1.5)	6.4 (1.5)	−0.4 (−1.4, 0.6)	5.5 (1.6)	5.1 (1.6)	0.31 (−0.75, 1.37)
FD	32.3 (9.2)	34.7 (8.7)	44.9 (12.9)	43.7 (12.9)	1.2 (−7.5, 9.9)	49.4 (8.3)	47.2 (8.3)	2.22 (−3.40, 7.83)
Total	47.9 (13.6)	46.8 (18.2)	69.6 (18.1)	67.3 (18.1)	2.3 (−9.8, 14.4)	73.2 (12.5)	67.7 (12.5)	5.51 (−2.83, 13.86)

*Abbreviations:* ST, sling exercise training group; CPM, continuous passive motion group; HSS score, Hospital for Special Surgery Knee Score; FlexDeform, flexion deformity; ROM, range of motion; SF-36, Short Form Health Survey; WOMAC score, Western Ontario and McMaster University Osteoarthritis Score; FD, functional difficulty.

*denotes a significant difference (*P* ≤ 0.025); ^**†**^denotes a statistical tendency (*P* ≤ 0.100). ^‡^ANCOVA: adjusted for baseline, sex, weight and age.

Values of the pretest are presented as mean (standard deviation). Values of the posttest and follow-up-measurement are presented as estimated marginal means (standard deviation).

## Discussion

The objective of the present randomized clinical study was to compare the effectiveness of a new sling-based ROM therapy with the traditional CPM application as an adjunct to daily physiotherapy following TKA. The knee joint mobilization in the sling requires the activation of muscles. Furthermore, the unstable support during the performance of ROM exercises might contribute to higher demands on muscle strength and muscle coordination. Therefore, it was assumed that an ST might be advantageous for early recovery following TKA.

There is evidence that ST has a significant positive, short-term effect on pFL of 6°. A medium-term benefit of these positive effects on knee flexion ROM could not be confirmed because no differences between groups were determined at the 3-month follow-up. Furthermore, there were no significant beneficial effects of ST on the secondary outcomes (aFL, aEX, pEX, pain, physical activity, static postural control, length of hospital stay and health-related status, function or quality of life).

A recent review on the effectiveness of adjunctive CPM therapy compared to physiotherapy alone reported short-term effects on aFL and pFL by 3° and 2°, respectively [1]. The authors suggested that these effects on ROM are too small to be clinically relevant. Taking into account the fact that the application of CPM is associated with high costs for the rental or acquisition of the device and additional technical and personnel efforts to set up and operate the machine, it was suggested that an additional ROM of more than 5° is required to justify its use [1]. Following this recommendation, we hypothesized a clinically relevant mean difference in ROM of 5° between ST and CPM therapy. Our present data demonstrate a between-group difference in pFL of 6°, which is above the hypothesized and clinically relevant difference. This result leads to the assumption that the application of an adjunctive ST therapy in the early postoperative phase could be recommended as a part of the rehabilitation program following TKA. Nevertheless, the clinical relevance remains uncertain as the confidence interval was fairly wide and some methodological limitations have to be considered.

### Study limitations

Our study was limited by the small sample size. The target number of participants was not achieved by the scheduled day of closure due to an unexpectedly low number of patients that met the inclusion criteria. It was not possible to extend the study to achieve adequate enrollment, as the date of termination of financial support of the project had been reached. The high proportion of ineligibility reduces the generalizability of the findings. A post-hoc power analysis was conducted for all secondary outcomes that did not reach statistical significance in order to rule in or rule out inadequate power as a threat to the internal validity of the findings [35]. The post-hoc power coefficients were low (power < 0.80). Thus, the study was underpowered for many outcome variables and a lack of power, possibly due to small sample size, is an alternative explanation of the statistically non-significant findings. It could be assumed that, if the study had been adequately powered we could have seen a greater number of statistically significant differences.

A second limitation was the 26% dropout rate at the 3-month follow-up. However, none of the patients withdrew from the study for a reason related to the study treatment. Missing values will result in a reduction of the number of cases for analysis which reduces precision and possibly introduces bias. The missing-data problem was handled by imputing missing values using multiple imputation [29,30].

A further limitation was the inability to blind the patients and practitioners, which was impossible due to the nature of the intervention. Although it would be appropriate, it was not possible to run a 3-arm trial including an arm with no additional intervention because CPM combined with usual physiotherapy is the standard treatment following TKA in the Department of Orthopedics at the University Medicine Rostock. Thus, it was not considered appropriate to exclude the CPM therapy.

Despite these limitations, a statistically significant and clinically relevant positive result relating to the ST therapy could be presented.

## Conclusions

This study was the first randomized controlled trial to systematically assess the effectiveness of a new active sling-based in-hospital ROM exercise program with the standard-of-care therapy (CPM) following TKA. Findings in the literature indicate that an adjunctive passive ROM therapy using CPM only slightly contributes to an initial regain of ROM. The present data suggest that more complex tasks using active sling-based exercises could have a further beneficial short-term effect on knee flexion ROM compared to conventional CPM therapy. A clinically relevant difference between groups was found in pFL. The ST is easy to carry out during hospital stay and is less expensive than CPM therapy. Since the cost-effectiveness could be increased while even improving the quality of clinical results, the application of an ST therapy in the post-operative phase can be recommended as part of the early post-operative rehabilitation management. Against the background of some methodological limitations, the results of the present study should be interpreted with caution and could be strengthened after a replication study is carried out on a larger sample set.

## Competing interests

The authors declare that they have no competing interests.

## Authors’ contributions

AMM participated in the conception and design of the study, acquisition, analysis and interpretation of data and drafting of the manuscript. MB participated in the conception and design of the study, interpretation of data and revising the manuscript critically for important intellectual content. SF participated in the acquisition and analysis of data. SB, RB and WM participated in the conception and design of the study and revising the manuscript critically for important intellectual content. All authors read and approved the final manuscript.
